# Sexual and contraceptive behaviour of young adults in Germany – Results from KiGGS Wave 2

**DOI:** 10.25646/9873

**Published:** 2022-06-29

**Authors:** Birte Hintzpeter, Laura Krause, Felicitas Vogelgesang, Franziska Prütz

**Affiliations:** Robert Koch Institute, Berlin Department of Epidemiology and Health Monitoring

**Keywords:** SEXUAL BEHAVIOUR, FIRST SEXUAL INTERCOURSE, CONTRACEPTION, MORNING-AFTER PILL, KIGGS WAVE 2

## Abstract

Sexual behaviour is an important aspect of sexual health. 18-year-old and older participants of the KiGGS cohort in KiGGS Wave 2 were asked about their sexual and contraceptive behaviour. Data from 2,966 women and 2,206 men were included in the analysis, which was adjusted to the age and sex distribution of the German population by means of weighting. More than half of the respondents report their first sexual intercourse before reaching the age of majority (women 61%, men 53%). Women report a lower age than men. With regard to the number of opposite-sex sexual partners in the last twelve months, almost 69% of women and 58% of men state that they have had contact. Three or more sexual partners were reported by 11% of women and 20% of men. 7.4% of women have same-sex and 1.4% have both same-sex and opposite-sex sexual contacts, among men the figures are 2.8% and 0.4%, respectively. When asked about the type of contraception used during the last sexual intercourse, about two thirds of the women and more than half of the men indicated the pill; a condom is used by about 44% of the women and about two thirds of the men. Almost one third of the women have already taken the morning-after pill. Overall, the results can help to support prevention and education campaigns on sexual and reproductive health.

## 1. Introduction

Sexual health is defined according to the World Health Organisation (WHO) in close connection and in line with the general concept of health [1]: ‘Sexual health is an integral part of overall health, well-being and quality of life. It is a state of physical, emotional, mental and social well-being in relation to sexuality, and not merely the absence of disease, dysfunction or infirmity [2]’. The different aspects of sexual health include sexual behaviour, sexual orientation and gender identity, as well as other aspects such as sexually transmitted infections (STI) [3].

Prerequisites for sexual health are a positive and respectful attitude towards sexuality and sexual relationships, and the opportunity to have pleasurable and safe sexual experiences, including freedom from violence and discrimination [2]. In addition to sexual self-determination, sexual education, sexual satisfaction and well-being, sexual health also includes the possibility to develop and live a sexual identity [4].

Sexuality is experienced differently in the different phases of life [5]. During adolescence, sexuality and sexual experiences are among the developmental tasks, along with coming to terms with one’s own body, detachment from one’s parents and forming social relationships [6]. Adolescence is associated with physical, psychological and emotional changes. The biological processes interact with the social context to affect the emotional and social development of the individual [7]. During adolescence, girls and boys have to deal with age-typical behavioural expectations and find appropriate strategies for dealing with them, this also applies to sexuality [8].

**Info box Sexual orientation**

A person’s sexual orientation describes whether they are romantically and sexually attracted to their own sex (homosexuality), the opposite sex (heterosexuality), both sexes (bisexuality) or neither sex (asexuality) [15].

The three dimensions of sexual orientation include sexual attraction or appeal (which genders a person is attracted to), sexual behaviour (with which gender they have sexual contact) and sexual identity [15]. Sexual identity is people’s fundamental self-understanding of who they are as sexual beings, how they perceive themselves and how they want to be perceived by others [18]. The three dimensions do not have to coincide; moreover, they can change over a lifetime [17].

The abbreviation LGBTIQ covers different sexual orientations and ways of living as well as gender identities: lesbian, gay, bisexual, transgender, intersex and queer people. Queer is a collective term that encompasses gender identities and sexual orientations that are not oriented towards the heterosexual gender binary. Younger LGBTI people in particular are more likely to describe themselves as queer [17].

Sexuality education is increasingly understood as a cross-sectional task in society. In addition to school and family, health and social services, the media and adult education are also involved [9]. The Federal Centre for Health Education (BZgA) has the legal mandate to develop concepts and media for sexuality education and to provide information on contraception. This is done with the participation of the federal states and in cooperation with representatives of the family counselling institutions of all providers [10]. The article Sexuality education for young people in Germany in this issue of the Journal of Health Monitoring uses data from the BZgA’s 2019 Youth Sexuality Study to show that young people use a variety of different sources and instances to obtain health information in this area. These include the teaching of knowledge and action at school, personal conversations, the internet or professional counselling in gynaecological practices and recognised counselling centres [11].

Sexuality is predominantly experienced in committed relationships in all age groups. Studies have shown that even in adolescence, relationships are often close, romantic and characterised by the ideals of love and fidelity [12]. Almost one fifth of girls and boys in Germany surveyed in the Health Behaviour in School-aged Children study (HBSC) 2017/18 had sexual intercourse at least once at the age of 15 years [13]. On average, girls are sexually active at an earlier age than boys [14]. Results of the German Health and Sexuality Survey (GeSiD), which was conducted by the University Medical Centre Hamburg-Eppendorf from 2018 to 2019, showed that gender differences also exist with regard to the number of opposite-sex sexual contacts. Heterosexual men report higher numbers of partners than heterosexual women. This is already evident for adolescents and young adults [15, 16].

According to the current state of research, three dimensions of sexual orientation (Info box) are distinguished, which do not have to coincide: sexual identity, sexual attraction or appeal, and sexual behaviour [15]. For example, a woman who has sex with women does not necessarily identify as lesbian or bisexual [17]. The different dimensions of sexual orientation are not rigid categories, but changeable phenomena that may change over a lifetime [19].

Contraceptive behaviour is also part of sexuality. Access to contraception is an important factor in enabling people to decide freely if, when and how many children they want to have [20]. Contraceptives include, for an example, hormonal contraceptives such as the pill, barrier methods such as the condom or diaphragm, the intrauterine device (IUD) and so-called natural methods of contraception. In addition to the contraceptive aspect, the condom also offers protection against STI [21].

More than 70% of the sexually active adult population uses contraception during sexual contact [22]. Reasons for not using contraception include a desire to have children or pregnancy [23]. In addition, there are couples who do not use contraceptives despite having no intention of becoming pregnant [24]. The morning-after pill is an emergency contraceptive that is mainly used after contraceptive mishaps or when contraceptives were forgotten. It is available without prescription in pharmacies since March 2015. Counselling is also offered here [25].

**KiGGS Wave 2**

Second follow-up to the German Health Interview and Examination Survey for Children and Adolescents

**Data owner:** Robert Koch Institute

**Aim:** Providing reliable information on health status, health-related behaviour, living conditions, protective and risk factors, and health care among children, adolescents and young adults living in Germany, with the possibility of trend and longitudinal analyses

**Study design**: Combined cross-sectional and cohort study

**Cross-sectional study in KiGGS Wave 2**

**Age range:** 0–17 years

**Population:** Children and adolescents with permanent residence in Germany

**Sampling:** Samples from official residency registries – randomly selected children and adolescents from the 167 cities and municipalities covered by the KiGGS baseline study

**Sample size:** 15,023 participants

**KiGGS cohort study in KiGGS Wave 2**

**Age range:** 10–31 years

**Sampling:** Re-invitation of everyone who took part in the KiGGS baseline study and who was willing to participate in a follow-up

**Sample size:** 10,853 participants

**KiGGS survey waves**

▶ KiGGS baseline study (2003–2006), examination and interview survey
▶ KiGGS Wave 1 (2009–2012), interview survey
▶ KiGGS Wave 2 (2014–2017), examination and interview survey

More information is available at www.kiggs-studie.de/english

This article presents results on the sexual and contraceptive behaviour of young adults, which were gathered as part of the second follow-up survey of the German Health Interview and Examination Survey for Children and Adolescents (KiGGS Wave 2). It thus ties in with the Robert Koch Institute’s (RKI) report Health situation of women in Germany, published at the end of 2020, which includes a focus chapter on sexual and reproductive health as well as one on girls’ health [17]. In KiGGS Wave 2, the participants of the KiGGS cohort who were already of age in the second follow-up survey (between 18 and 31 years) were also asked about their sexual behaviour. In addition to questions about age at first sexual intercourse, the number of sexual partners was asked, as well as questions about contraceptives and emergency contraception.

The results can help to support prevention and education campaigns on sexual and reproductive health, for example to adapt information materials on sexuality education and contraception to specific target groups. In addition, they can contribute to the evaluation of measures, complement the results of existing studies in this area and thus contribute to the scientific discourse.

## 2. Methodology

### 2.1 Sample design and study conduct

The basis for the analyses in this article are the data from the KiGGS cohort. The KiGGS baseline survey, which was conducted by the RKI from 2003 to 2006, for the first time provided population-based, nationally representative results on the health situation of 0- to 17-year-old children and adolescents in Germany. Within the framework of the KiGGS cohort, these children and adolescents will be further observed. The KiGGS baseline survey was followed by two further waves. After KiGGS Wave 1 (2009 to 2012), KiGGS Wave 2 (2014–2017) provides the most recent data until now [26]. Participants in the KiGGS baseline survey who were still available and willing to participate again were invited back to the study. At the time of KiGGS Wave 2, a total of 10,853 cohort participants aged 10 to 31 years could be interviewed again; the re-participation rate was 62%. A detailed description of the KiGGS cohort can be found elsewhere [27, 28].

The present analyses are based on data from 5,172 young adults (2,966 women and 2,206 men) who were between 18 and 31 years old in KiGGS Wave 2 and had valid information on sexual and contraceptive behaviour.

### 2.2 Operationalisation of variables

#### Sexual and contraceptive behaviour

In KiGGS Wave 2, the adult participants in the KiGGS cohort were asked questions about sexual and contraceptive behaviour for the first time. The following questions about sexual behaviour are part of the analyses: ‘How old were you when you had sex for the first time?’ (open answer field to indicate age) and ‘How many sexual partners did you have in the last 12 months?’. As an answer to the last question, both the number of women and men should be given. In addition to determining the number of sexual partners in the last twelve months (‘none’, ‘one’, ‘two’, ‘three’ and ‘more than three’), the proportion of respondents with at least one sexual partner of the same and/or opposite sex in the last twelve months could be generated from this. The following questions were asked about contraceptive behaviour: ‘Are you currently using contraceptives?’ (response categories: ‘yes’, ‘no’), ‘Which contraceptives did you or your partner use during the last sexual intercourse?’ (‘birth control pill’, ‘condoms’, ‘diaphragm’, ‘chemical contraceptives’, ‘IUD’, ‘natural methods’, ‘other’, ‘none’), ‘Do you use condoms during sexual intercourse?’ (response categories: ‘yes’, ‘no’), ‘Have you ever taken the birth control pill?’ (response categories: ‘yes’, ‘no’) and ‘Have you ever taken the morning-after pill?’ (response categories: ‘yes’, ‘no’).

#### Education, migration-related characteristics and family type

In KiGGS Wave 2, respondents indicated their highest level of education. The International Standard Classification of Education (ISCED-11) was used to classify the data. The education categories were divided into a low, a medium and a high education group [29].

The migration status is determined on the basis of the information on the country of birth of the participants as well as the country of birth and the nationality of the parents. Participants who migrated to Germany themselves or whose two parents were not born in Germany or are not German citizens are considered migrants. Another migration-related characteristic is the language spoken at home (exclusively German, other language/s) [30]. With regard to a partnership, the question was asked in KiGGS Wave 2 whether the respondents lived with a partner in a joint household (response categories: ‘yes’, ‘no’).

### 2.3 Statistical methods

For the descriptive analyses, prevalences with 95% confidence intervals were calculated in each case. The question on age at first sexual intercourse was already asked of all cohort participants aged 14 and older.

For this reason, the data basis for the analyses refers to all persons between 14 and 31 years (n=4,639 girls and women, n=3,870 boys and men). In order to take into account the right censoring of the data for the age at first sexual intercourse, i.e. the different ages of the participants at the time of KiGGS Wave 2, survival analyses were used. Survival analyses take into account that a person who is only 17 years old, for example, cannot give any information about a possible future event at the age of 19 or 20.

The data on age at first sexual intercourse are extrapolated by this procedure to the case where the complete KiGGS cohort would have been followed up to the age of 31. Gender differences between the curves were tested using a log-rank test in SAS.

The analyses were carried out with a weighting factor that both removes the drop-out from the baseline survey and adjusts the population figures by age, sex and education to the current survey date (31.12.2015). A statistically significant difference is assumed if the p-value is smaller than 0.05.

The analyses were conducted using the survey procedures of Stata 17.0 (Stata Corp., College Station, TX, USA, 2015) in order to take the cluster design of KiGGS and the weighting appropriately into account when calculating confidence intervals and p-values. Analyses on first sexual intercourse were conducted using SAS 9.4 (SAS Institute, Cary, NC, USA).

## 3. Results

**Figure 1:**
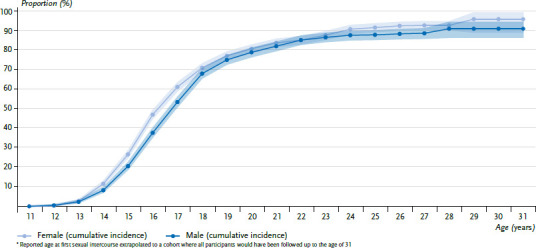
Reported age at first sexual intercourse^*^ among 14- to 31-year-olds by sex, cumulative incidence (n=4,639 girls and women, n=3,870 boys and men) Source: KiGGS Wave 2 (2014–2017)

First, the anaylses on the age of first sexual intercourse are regarded. For this purpose, persons between 14 and 31 years of age were considered. Of the adolescents and young adults participating, about one in four girls or women (26.6%) and one in five boys or men (20.6%) reported having had their first sexual intercourse by the age of 15. More than half of the respondents report their first sexual intercourse before reaching the age of majority (61.0% of girls and women and 53.3% of boys and men). About one in five people had not yet had sexual intercourse by the age of 20. Until the age of 30, it is about 4% of women and 9% of men (Figure 1). Girls and women report a lower age at first sexual intercourse than boys and men (the gender difference is statistically significant, p<0,001).

When asked about sexual contacts in the last twelve months, about 10% each of women and men report having had no contacts (Table 1). More than two thirds of women (68.8%) and more than half of men (57.8%) report exactly one sexual contact, while about 10% of women and 12% of men report two contacts. About 11% of women and about 20% of men report having had three or more sexual contacts.

Table 2 differentiates between same-sex and opposite-sex sexual partners. Over 90% of the interviewed women and men report opposite-sex sexual contacts in the last year before the survey. Among women, 7.4% have same-sex and 1.4% have both same-sex and opposite-sex sexual contacts. The corresponding proportions for men are 2.8% and 0.4 % respectively. It should be noted that the analyses on same-sex and opposite-sex partners are based on very small case numbers (Table 2).

In terms of contraceptive behaviour, 76.5% of women and 59.1% of men reported currently using contraceptives at the time of the survey. Further analyses show that women and men living in a stable partnership use contraception significantly more often than women and men without a stable partnership (women 79.2% v. 68.2%, p≤0.001 and men 63.1% v. 52.9%, p≤0.001). Around two-thirds of women (67.5%) and about half of men (51.0%) report that they are currently living in a committed partnership (data not shown).

**Figure 2:**
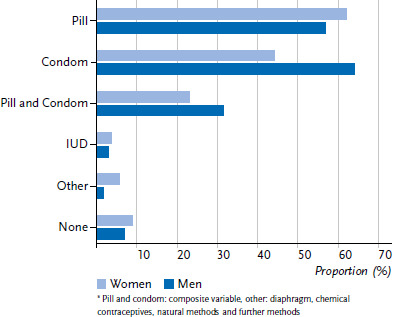
Type of contraceptive^*^ used during last sexual intercourse (data in percent, multiple answers possible) among 18- to 31-year-olds with sexual intercourse experience by sex (n=2,880 women, n=2,160 men) Source: KiGGS Wave 2 (2014–2017)

When asked about the type of contraception used during the last sexual intercourse, it becomes apparent that the pill and the condom were used most frequently. More than half of the women (62.1%) and men (57.0%) report using the pill. A condom is used for contraception by 44.1% of women and 64.2% of men. The combined use of pill and condom is reported by 23.1% of women and 31.6% of men. The IUD, on the other hand, is used much less frequently: 3.8% of women and 3.0% of men report it as the contraceptive method used. Other contraceptives such as the diaphragm, chemical contraceptives or natural methods also play a minor role. 8.9% of women and 6.8% of men say they did not use contraception during their last sexual intercourse (Figure 2).

Furthermore, the participants of the KiGGS cohort were asked whether they generally use condoms during sexual intercourse. Condoms are generally used by 27.3% of the women during sexual intercourse, about one third of the women (32.2%) uses condoms occasionally, 40.5% of the women do not use condoms. For men, the proportions of basic (41.8%) and occasional use (34.6%) are higher. Slightly less than a quarter of men (23.6%) does not use condoms. If only men who do not live in a committed partnership are considered, 59.9% of them report that they use condoms in principle, 33.3% use condoms occasionally and 6.7% of them do not use condoms. In addition, the interviewed women were asked whether they had ever taken the pill. A majority of them (92.6%) answered this question in the affirmative (data not shown).

In addition to taking the pill, participants were also asked about emergency contraception (using the morning-after pill). Almost one third of the women (30.8%) had experience with taking the morning-after pill. Stratified analyses according to age, education and migration status show no statistically significant differences. This also applies if the language spoken at home is considered as another migration-related characteristic (Table 3).

## 4. Discussion

Data on the sexual and contraceptive behaviour of young adults from KiGGS Wave 2 show that about half of the participants experienced their first sexual intercourse before reaching the age of majority; among women it is 61%, among men 53%. This finding is consistent with the data from the eighth wave of the Youth Sexuality Study by the BZgA, which is based on a survey of 14- to 25-year-olds from 2014. According to this, 39% of adolescents are sexually active for the first time at the age of 16, and among 17-year-olds the proportion is 58% [16]. According to the KiGGS Wave 2 data, about every fourth girl and every fifth boy have their first sexual intercourse by the age of 15. The proportions are therefore roughly comparable to those of the HBSC study, in which 15-year-olds provide information on whether they have already slept with someone. In the 2013/14 HBSC study, this applied to 19.6% of girls and 22.3% of boys [31], in 2017/18 the proportions were 16.7% (girls) and 19.7% (boys) [13].

When making the comparison, however, it should be noted that the questions on the age of first sexual intercourse in KiGGS Wave 2 were collected retrospectively, so that a recall bias cannot be excluded. Since data on sexuality have only been collected once in the KiGGS study so far, no information on trends can be made. Results of the ninth wave of the Youth Sexuality Study from 2019 show that the proportion of adolescents who are younger than 17 years of age at first sexual intercourse has been declining for several years. This continues the trend that young people are becoming sexually active later and later [32]. About 20% of the respondents had not had sexual intercourse by the age of 20 – this proportion is higher than the 16% found in the Youth Sexuality Study [16], at the age of 30 it is about 4% of women and almost 9% of men. The lack of the right partner or cultural reasons could play a role in sexual restraint [16].

The majority of 18- to 31-year-old participants from KiGGS Wave 2 (almost 69% of women and 58% of men) had opposite-sex sexual contacts with exactly one person in the last twelve months. This indicates that a high proportion of young adults are in a committed relationship. In the study, about two-thirds of young women and about half of young men stated they were currently in a committed relationship. This is consistent with studies that have shown that, already in adolescence, sexuality is predominantly lived in committed relationships. Relationships are often close, romantic and characterised by the ideals of love and fidelity [33]. Being single is usually seen as a temporary phase between two relationships, which is often spent sexually in a rather restrained way. In a serial monogamous relationship pattern, new firm and faithful relationships are constantly entered into [14].

About 10% of the young women and men had no sexual contacts in the last twelve months before the survey, about 10% of the women and almost 12% of the men had sexual contacts with two persons. About 20% of the men – and thus almost twice as many as women (about 11%) – stated that they had sexual contacts with three or more persons. A similar picture emerges from a cross-sectional study of 654 students at the Technical University of Dresden in 2012, in which, among other things, sexual risk behaviour was examined: Of the sexually active students, 4% reported none, 53% one, 14% two, 10% three to nine and 1% ten to 15 sexual contacts in the last twelve months [34]. Our analyses are only comparable with the Youth Sexuality Study to a limited extent, since these refer to the total number of previous sexual partners [16].

Various European sex surveys also found that men report a higher number of sexual contacts than women [35, 36]. Heterosexual men report higher numbers of partners than heterosexual women [15]. The reason given is, among other things, a different response behaviour. Men tend to present themselves as sexually experienced and active due to social expectations. They may therefore indicate a higher number of female partners. Estimation errors among men with many sexual partners could also play a role. Another reason given is that men may have sex more often with women who systematically do not participate in surveys, such as sex workers [15].

According to the data from KiGGS Wave 2, 7.4% of young women and 2.8% of young men had same-sex sexual contacts in the last twelve months. Both same-sex and opposite-sex sexual contacts were reported by 1.4% of female and 0.4% of male participants. Results from the 2018 to 2019 GeSiD survey show that 15% of 18- to 35-year-old women had at least one sexual experience with another woman. For men of the same age, the figure is 7.4% [15]. Again, it should be noted that these results refer to experiences ever made. In contrast, the KiGGS study refers to the last twelve months.

When interpreting the data, it must be taken into account that same-sex sexual contacts do not necessarily have to be associated with a homosexual identity (Info box Sexual Orientation). Overall, according to data from the GeSiD study, 0.9% of women and 1.8% of men define themselves as homosexual and 1.8% of women and 0.9% of men as bisexual [15]. Slightly higher figures are found in the Youth Sexuality Study from 2019. An orientation other than purely heterosexual is more likely to be reported by female than by male respondents: 2% of 14- to 25-year-old women report being homosexual, 8% identify as bisexual, among men it is 3% [32]. The fact that (young) women report at least one same-sex sexual experience comparatively frequently could be due to a generally greater social openness towards same-sex intimacy and sexuality of women, which contributes to a greater scope for experience and thus also for answers in surveys [37].

For contraception during the last sexual intercourse, those participating in KiGGS Wave 2 most frequently used the pill and the condom (pill: women 62%, men 57%; condom: women 44%, men 64%). The fact that the pill and the condom are the most important contraceptives in Germany was also shown in the 2018 BZgA study on contraceptive behaviour of adults: 47% of women and 48% of men name the pill as their current contraceptive method. In the case of condoms, it is 37% of women and 56% of men. Compared to previous waves of the study, the condom is used significantly more often as a contraceptive. From 2011 to 2018, there was an overall increase from 37% to 46%. During this time, the proportion of women taking the pill decreased from 53% to 47%. There has been a sharp decline in use of the pill particularly among 18 to 29 year olds, from 72% to 56% [22]. A decline in pill use was also reported in the waves of the 2015 [16] and 2021 Youth Sexuality Study [32]. This can be observed especially among sexually active girls between 14 and 17 years of age. The reason given is a rather critical attitude towards hormonal contraceptive methods. This could be related to a general change in the perception of norms, such as an increase in health awareness [32]. Especially in social media, the concern to live healthier and more naturally, also with regard to contraceptive behaviour, is a topic of discussion [38]. In KiGGS Wave 2, about 93% of women stated that they had ever taken the pill. In contrast, the proportion of women who used the pill during their last sexual intercourse is 62%. This result could also indicate a decline in pill use. With regard to contraception during the last sexual intercourse, the IUD plays a subordinate role in the present analyses. This finding was also shown in the BZgA study ‘frauen leben 3’. According to this study, the use of the IUD increases over the course of life. Women over the age of 40 are the main users of IUDs as a contraceptive [39].

About 42% of the men always use condoms during sexual intercourse, about one third uses condoms occasionally. Slightly less than a quarter of the men says they do not use condoms. Almost 7% of men without a stable relationship report that they generally do not use condoms. This result is lower than that reported in the GeSiD study, according to which 22% of 18- to 79-year-old men who are currently single have never used a condom during sexual intercourse in the past year [40]. In this comparison, however, the different survey times, questions and age groups should be considered.

The use of emergency contraception could also be examined with the data from KiGGS Wave 2. Accordingly, 30.8% of the 18- to 31-year-old women have ever taken the morning-after pill. This result is comparable to the data from the Youth Sexuality Study 2019, in which 27% of 14- to 25-year-olds stated that they have used the morning-after pill before, including 9% more than once. Among 18- to 25-year olds, it is 29% (20% reporting single use, 9% multiple use) [32]. Our results show no significant differences according to age, education or migration status. As a further migration-related characteristic, the language spoken at home was included in the analyses in order to map possible language barriers to information materials. There were no significant differences for this variable either. However, the proportions of participants with migration-related characteristics are relatively small in relation to the comparison groups.

The results indicate that the use of emergency contraception is independent of sociodemographic factors. The Youth Sexuality Study 2015 points in the same direction by showing that respondents who had their first sexual intercourse with a trusted partner or for whom contraception was discussed in detail at home also used the morning-after pill (single use 15%, multiple use 6%) [16]. This is followed by the results of the Youth Sexuality Study 2019, which show that knowledge about the morning-after pill is almost universal among the girls and young women surveyed [32]. The prescription requirement for the morning-after pill was lifted in March 2015 to facilitate access to this emergency measure. Since then, there has been a significant increase in usage. According to data from the Federal Union of German Associations of Pharmacists, sales figures in self-medication have risen sharply since 2015, but there has been a significant decline in medical prescriptions of the morning-after pill. Since 2015, when 662,000 packs were dispensed, the number has risen steadily to a total of 877,000 packs in 2019. In 2020, there was a decrease to 848,000 packs [41]. The available data indicate use in all social groups and emphasise the need to ensure low-threshold access options.

As a limitation to the present analyses, it must be taken into account in the interpretation that the self-reports were collected retrospectively. It cannot be ruled out that the results may be distorted by socially desirable response behaviour or that there is a memory bias, i.e. participants no longer remember events correctly or subsequently attach more or less importance to events than they originally did.

Overall, the data on sexual and contraceptive behaviour from KiGGS Wave 2 provide a further data basis focusing on young adulthood in addition to the established monitoring data from the BZgA, the data on sexual and contraceptive behaviour from the HBSC study and the data from the GeSiD study. The present analyses confirm and complement the results of the studies mentioned, such as the calculations of cumulative incidences of first sexual intercourse or the analysis of sociodemographic factors influencing the use of the morning-after pill. Here it could be shown that the utilisation takes place in all social groups; belonging to a certain educational or population group does not seem to have any influence. This suggests that sexuality education information is reaching young adults.

The data on sexual and contraceptive behaviour from KiGGS Wave 2 also show potential for further analyses, as extensive co-variables are available in the cohort approach. Thus, correlation analyses with various demographic characteristics are possible, as exemplified in an article on the utilisation of outpatient gynaecological services [42]. With regard to longitudinal analyses, the indicators of sexual and reproductive health in young adulthood can also be used as outcome variables, for example in connection with mental health problems in childhood or adolescence [43]. For future surveys and analyses, the impact of the COVID-19 pandemic – including the containment measures – on sexual health will also play a role, for example with regard to consequences for partnership relationships and sexual contacts. Media narratives on sexuality-related changes due to the COVID-19 pandemic could be identified, but empirical data are still lacking [44].

## Key statement

- More than half of the respondents had their first sexual intercourse before reaching the age of majority, 61% of women and about 53% of men.
- Almost 69% of women and 58% of men had exactly one opposite-sex sexual partner in the last twelve months, three or more were reported by 11% of women and 20% of men.
- Same-sex sexual contacts were reported by 7.4% of women and 2.8% of men, both same-sex and opposite-sex sexual contacts were reported by 1.4% of women and 0.4% of men.
- During the last sexual intercourse, the most common form of contraception was the pill or a condom. Around one third of the women stated that they had already taken the morning-after pill.

## Figures and Tables

**Table 1:** Number of opposite-sex sexual partners in the last twelve months among 18- to 31-year-olds with sexual intercourse experience by sex (n=2,950 women, n=2,206 men) Source: KiGGS Wave 2 (2014–2017)

Number of sexual partners	Women		Men	
	%	(95% CI)	%	(95% CI)
None	10.4	(8.9–12.0)	10.2	(8.5–12.2)
1	68.8	(66.4–71.2)	57.8	(54.7–60.8)
2	10.2	(8.6–12.0)	11.7	(10.0–13.6)
3	4.8	(4.0–5.8)	7.9	(6.4–9.7)
> 3	5.8	(4.6–7.3)	12.4	(10.4–14.7)

CI=confidence interval

**Table 2:** At least one sexual partner of the same and/or opposite sex in the last twelve months among 18- to 31-year-olds with sexual contacts by sex (n=2,800 women, n=2,027 men) Source: KiGGS Wave 2 (2014–2017)

At least one…	Women			Men		
	%	(95% CI)	n	%	(95% CI)	n
opposite-sex sexual partner	94.0	(92.5–95.1)	2,654	97.7	(96.6–98.4)	1,979
same-sex sexual partner	7.4	(6.0–9.0)	182	2.8	(1.9–3.9)	55
same and opposite-sex sexual partner	1.4	(0.8–2.5)	36	0.4	(0.2–1.1)	7

CI=confidence interval

**Table 3:** Use of emergency contraception (ever taken the morning-after pill) (data in percent) among 18- to 31-year-old women with sexual intercourse experience (n=2,961) Source: KiGGS Wave 2 (2014–2017)

	%	(95% CI)	n
Total	30.8	(28.4–33.4)	838
**Age group**			
18–24 years	29.0	(26.1–32.1)	501
25–31 years	32.6	(28.8–36.6)	337
**Education**			
Low education group	24.5	(17.8–32.8)	73
Medium education group	31.1	(28.1–34.1)	517
High education group	33.6	(28.9–38.6)	231
**Migration status**			
No	30.0	(27.5–32.5)	742
Yes	34.4	(27.0–42.7)	91
**Language spoken at home**			
German only	30.5	(28.0–33.1)	728
Other language/s	32.8	(25.8–40.6)	110

CI=confidence interval
